# Optimization of the seat position for a personal vehicle equipped with a crankset: pilot study

**DOI:** 10.1038/s41598-024-56446-y

**Published:** 2024-03-09

**Authors:** Łukasz Bereś, Justyna Pyrzanowska, Dagmara Mirowska-Guzel, Marcin Obszański, Paweł Pyrzanowski

**Affiliations:** 1 Institute of Aeronautics and Applied Mechanics, Warsaw University of Technology, Nowowiejska str. 24, 00-665 Warsaw, Warsaw Poland; 2 Medical University of Warsaw, Żwirki i Wigury str. 61, 02-091 Warsaw, Warsaw Poland

**Keywords:** Mechanical engineering, Ecology, Medical research

## Abstract

The aim of the study was to optimize the seat for a personal vehicle equipped with a crankset mechanism, meant for everyday use. The inclination of the seat backrest was selected on the basis of theoretical considerations. Then dynamic tests were carried out on a group of young, healthy men in order to verify the ergonomic aspects of the seat position in relation to the crankset and determine the efficiency of the human-mechanism system with a load of 50 W. The data obtained from the dynamic tests were subject to statistical analysis. Research has shown that higher seat positions result in statistically higher efficiencies. In addition, a holistic analysis of the personal vehicle design problem shows that the upper position of the seat is also the best. The results of the research can be used to optimize personal vehicles using human force as a drive.

## Introduction

The overall purpose of the research was to optimize the seat for a vehicle equipped with a crankset mechanism.

The history of a typical, commonly used, 2-wheeled bicycle, equipped with a crankset, basically began around the nineteenth century^1^. Since then, countless bicycle variations have been developed. A characteristic feature of almost all designs is a crankset mechanism equipped with pedals. As novelties such as steel, tires, bicycle chain, derailleur system and others appeared over time, they found their way into bicycle design. In recent years, the price of electric motors has dropped significantly and will continue to fall^2^, which opens a new chapter in the history of vehicles and opened the way to the development of a personal vehicles.

The use of human muscle power in individual transport is often an attractive solution compared to motor vehicles (cars, motorbike) but mainly for short distances (several kilometres). A hybrid bike, i.e. a motor-assisted bike, can be successfully used on longer routes (several dozen kilometres), though. Yet, a human muscle powered individual vehicle is particularly advantageous, because a human being is generally adapted to moving their body. Basically, the idea behind an individual vehicle is to match a person’s energy load to their natural capabilities. Accommodating 2 to 4 people in a human muscle powered vehicle significantly increases the weight of such a vehicle, causing greater resistance to vehicle movement, which may lead to the human body being overloaded.

The use of wheels significantly reduces the resistance to movement compared to a typical walk or run, so it is a particularly effective way of getting around. A personal vehicle can exist without motor support, but with a motor it creates a very interesting solution, whose functional features can be similar to the commonly known microcars^3,4^. Individual transport can partly replace the commonly used 5-passenger cars, which are often used by 1 person, and additionally, combined with smart technologies^5,6^, eliminate traffic jams during peak hours. In addition, personal vehicles can be produced at a lower price than a standard microcar, while maintaining most of the utility features, which is expected to have a positive impact on the development of less developed regions by increasing access to transport for more people.

The research was aimed at estimating how ergonomics and the human factor affect the global design of the personal vehicle equipped with a crankset and how important it is. This research was conducted because these are optimization factors that are difficult to derive by theoretical considerations^7^. An attempt was made to examine whether there is any specific seat position in relation to the crankset that maximizes the efficiency of the human-mechanism system and whether it is related to ergonomics, or perhaps it is individual for each person. The data collected from the tests can be used to perform any optimization where the goal is to cover the planned distance with less energy expenditure.

The personal vehicle that has been considered in this article is a vehicle that is generally intended to be used by one person only. It is a light, 3 or 4-wheeled vehicle with a track width of approximately 0.8 m (a human’s width), generally built-up, protecting a human against weather conditions and intended for moving on paved roads. The curb weight of the vehicle, depending on the materials used for construction and the mounted vehicle equipment, may vary from 30 to 130 kg. The basic components of such a vehicle are the frame, seat, wheels, drive system, steering system, cover and brake system. Additional equipment may include lamps, counters, battery, booster motor or motors, windshield cleaning system, gearshift system, heating and cooling system, trunk, vehicle locking system. In addition, it is a vehicle for everyday use.

**Figure 1:**
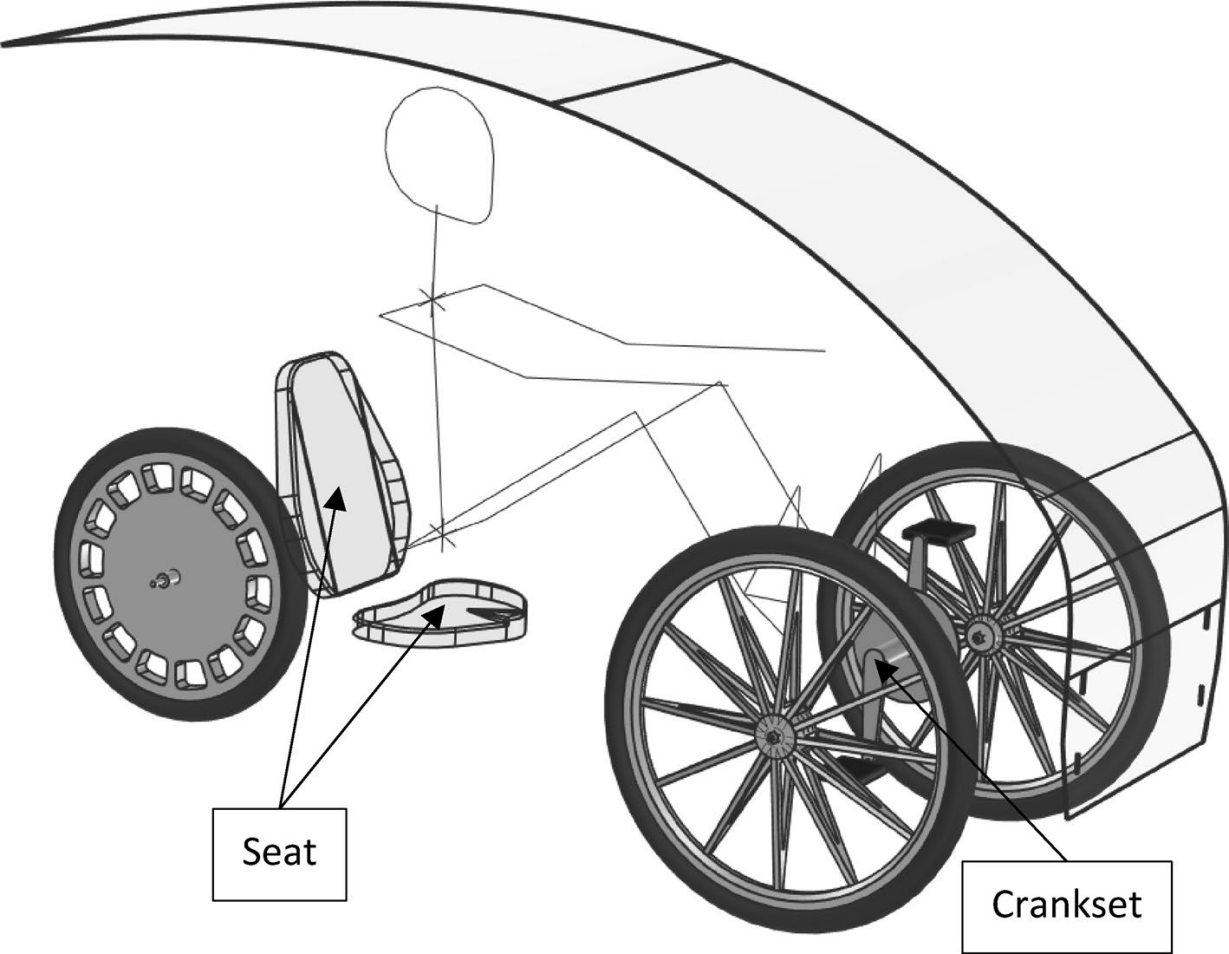
The outline of a three-wheeled personal vehicle equipped with the crankset.

**Figure 2:**
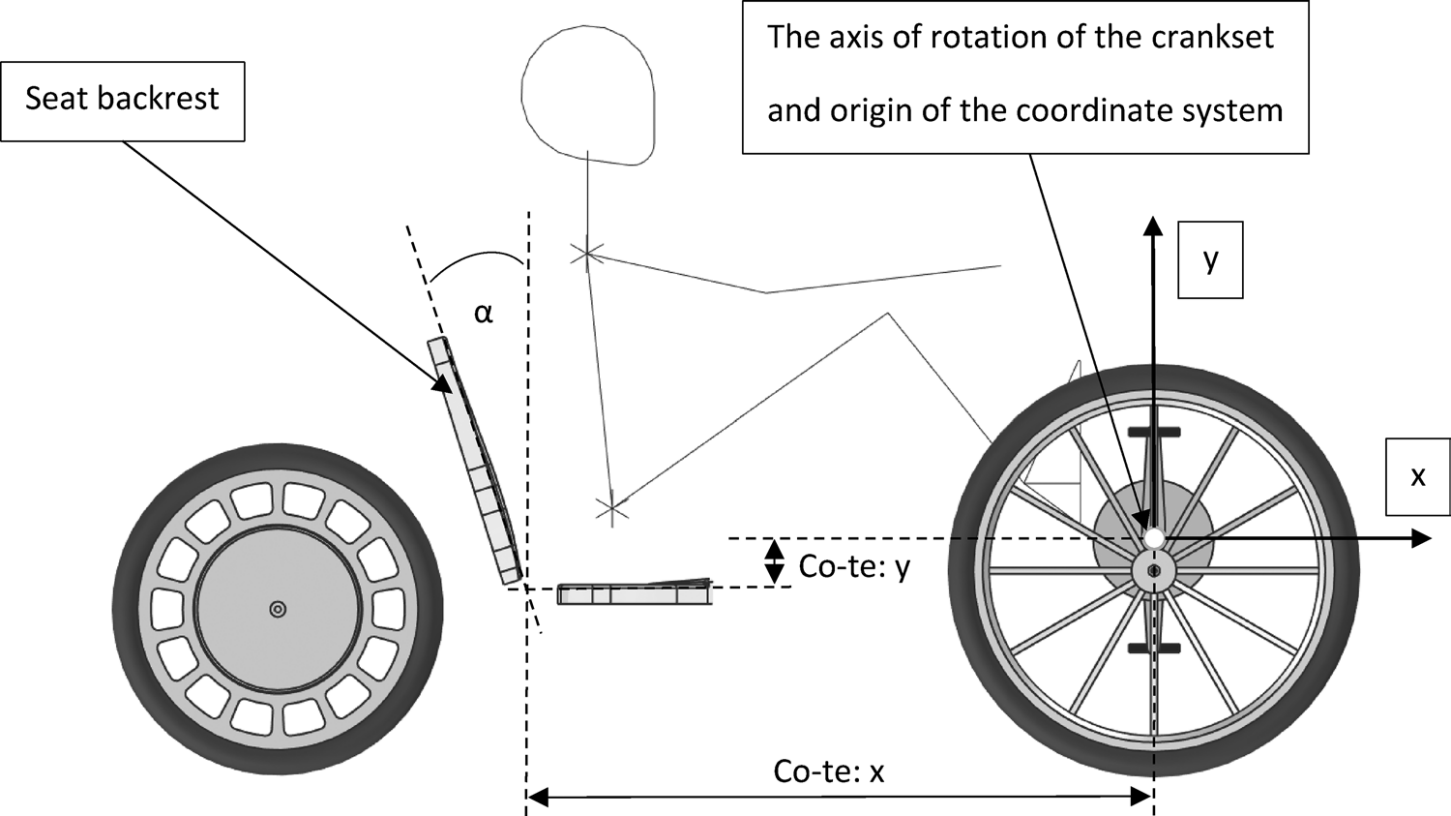
Parameters (α, x, y) of the seat in a personal vehicle that were considered during the tests.

The inclination of the seat backrest was selected on the basis of theoretical considerations. The efficiency of the human-mechanism system was tested depending on the position of the seat in relation to the crankset. In addition, after each test, the test person was asked to assess the ergonomic aspects of various positions of the seat. The research was basically conducted for use in personal vehicles used in road transport, but the results of the research can also be applied in the area of water transport. The outline of the personal vehicle equipped with a crankset is shown in Fig. 1. The variables describing the position of the seat that were the subject of the research are shown in Fig. 2.

The research makes a significant contribution to the development of personal vehicles that fit into the new global trend of the so-called all-weather bike. In vehicles of this type, a reclining driver’s position and minimization of the frontal area are usually considered. These ultimately force the seat to be positioned as low as possible. On the other hand, there are opinions about the difficulty in observing the road and the uncomfortable position while pedalling. The literature review showed that there are several studies into similar topics, but these are either theoretical considerations^8,9^ or research relating to maximizing performance mainly for cycling races^10^. The novelty of this study lies in the fact that it was carried out on a significant number of people exercising at a low power of 50 W, and so it checks the efficiency of the human-mechanism system and assesses ergonomics. In addition, theoretical considerations take into account the factor of movement resistance and the user’s visibility of the road ahead. To sum up, the research took into account all key factors affecting the position of the seat for the concept of personal vehicles for everyday use.

In designing the living environment for human beings, more and more attention is paid to the quality of human life^11–14^. Personal vehicles can contribute to an increase in interest in pro-ecological and health-promoting means of transport^15–17^. A human being generally needs some physical effort to maintain health. It is not recommended to overload the body, especially every day, because the muscle recovery time may be longer than 24 h^18^. Often, the environment around people forces them to be sedentary^19^, so designing the environment to force people to move can bring many benefits to society as a whole. In addition, low-power exercises can be a method of recovery^20^, and the personal vehicles supported, for example, with electric motors, can facilitate this task. Personal vehicles promote a healthy lifestyle and can significantly reduce lifestyle diseases related to lack of movement^21,22^. In general, exercise has a very positive effect on health^23,24^, as does cycling^25,26^. Moreover, it is now recognized that exercise has a much greater impact on health and longevity than genetics^27^.

Given the potential for personal vehicles in the future, even a small improvement can have a big impact on the environment. Considering the amount of energy used for individual transport^28^, personal vehicles seem to respond to the need to save energy and care for the environment.

## Material and methods

The research was divided into three stages. The first stage was selection of the seat backrest inclination, which was made on the basis of a theoretical analysis. The second stage was dynamic research on a group of people. During the dynamic tests, the position of the seat relative to the crankset was changed. The next stage of the research was a statistical analysis carried out on the data collected during the dynamic tests. Theoretical analysis made it possible to define the angle of inclination (α, see Fig. 2) of the seat backrest. The dynamic tests and statistical analysis made it possible to examine the influence of the position of the seat (x, y, see Fig. 2) relative to the crankset on the efficiency of the human-mechanism system.

### Theoretical analysis (selection of the seat backrest angle)

The seat with a horizontal base and a fixed backrest inclined at α = 17.5° was used for the tests. This type of seat is commonly used in stationary exercise bikes. Such an inclination is not accidental and is the result of much work on the ergonomics of the seat^29^. The inclination of α = 10–20° permits relieving the lumbar spine^30^ and enables undisturbed work of internal organs^31^, as it is for α = 0°, so it allows long-term and comfortable sitting. Also, the inclination in the range of α = 10–30° is beneficial, especially when vertical vibrations affect the person^32^, which in the case of personal vehicles will be caused by some unevenness on the road. In turn, the inclination α ≥ 30° may adversely affect the assessment of the road situation by the person driving the vehicle. Ultimately, the inclination of the seat backrest equal to α = 17.5° seems to be a good, ergonomic solution, because it is an average value that relieves the lumbar spine, enables uninterrupted work of internal organs, and minimizes the impact of vertical vibrations on humans.

### Dynamic research (assessment of ergonomic aspects and determination of efficiency)

The first objective of the dynamic tests was to check how the seat position affects the ergonomics of using the crankset mechanism. The second objective was to determine the gross and net efficiency for the human-mechanism system. In other words, it was analysed how the energy expended by humans is converted into mechanical energy that can be used in transport.

**Figure 3:**
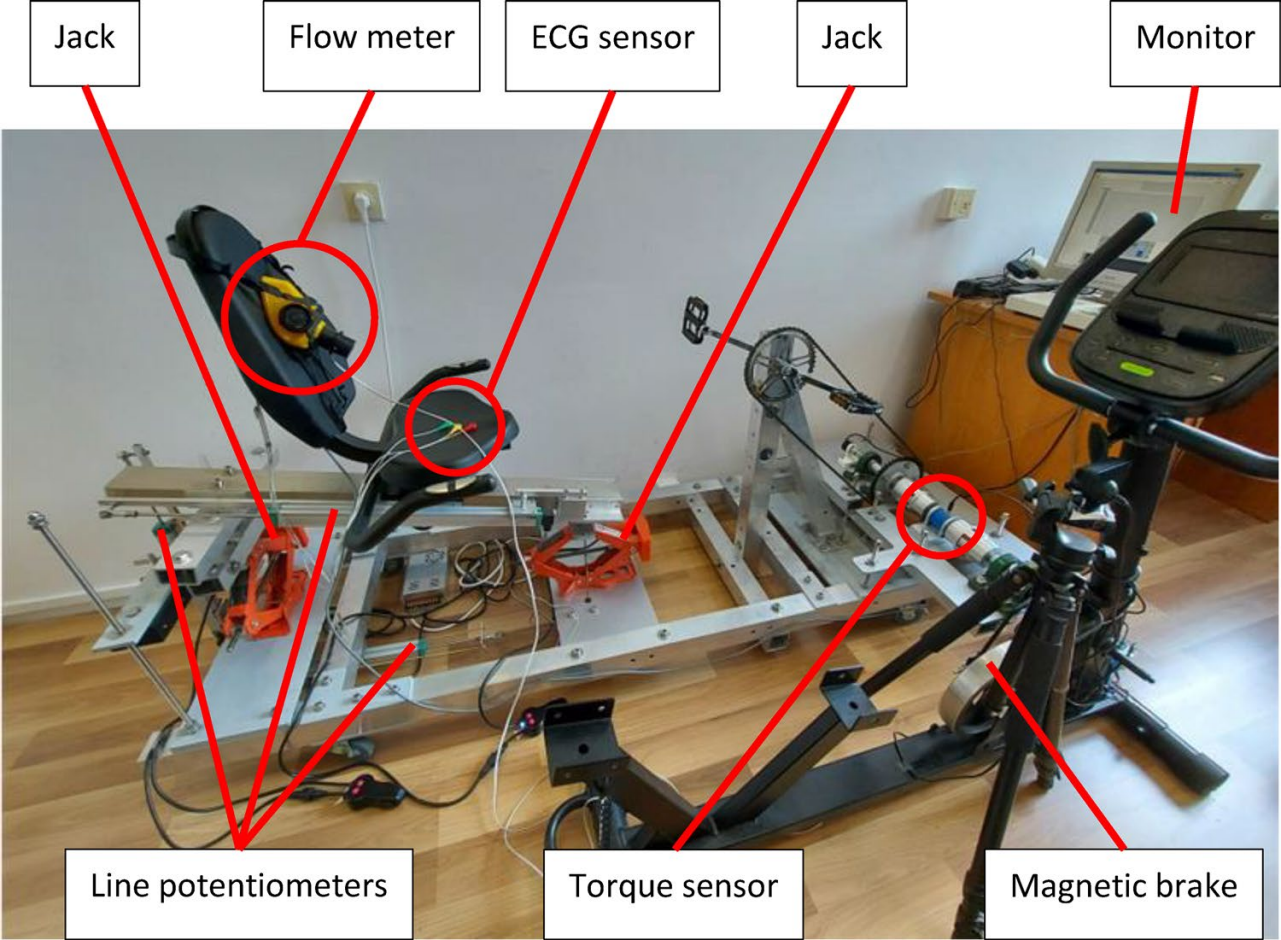
Stand for dynamic tests.

**Figure 4:**
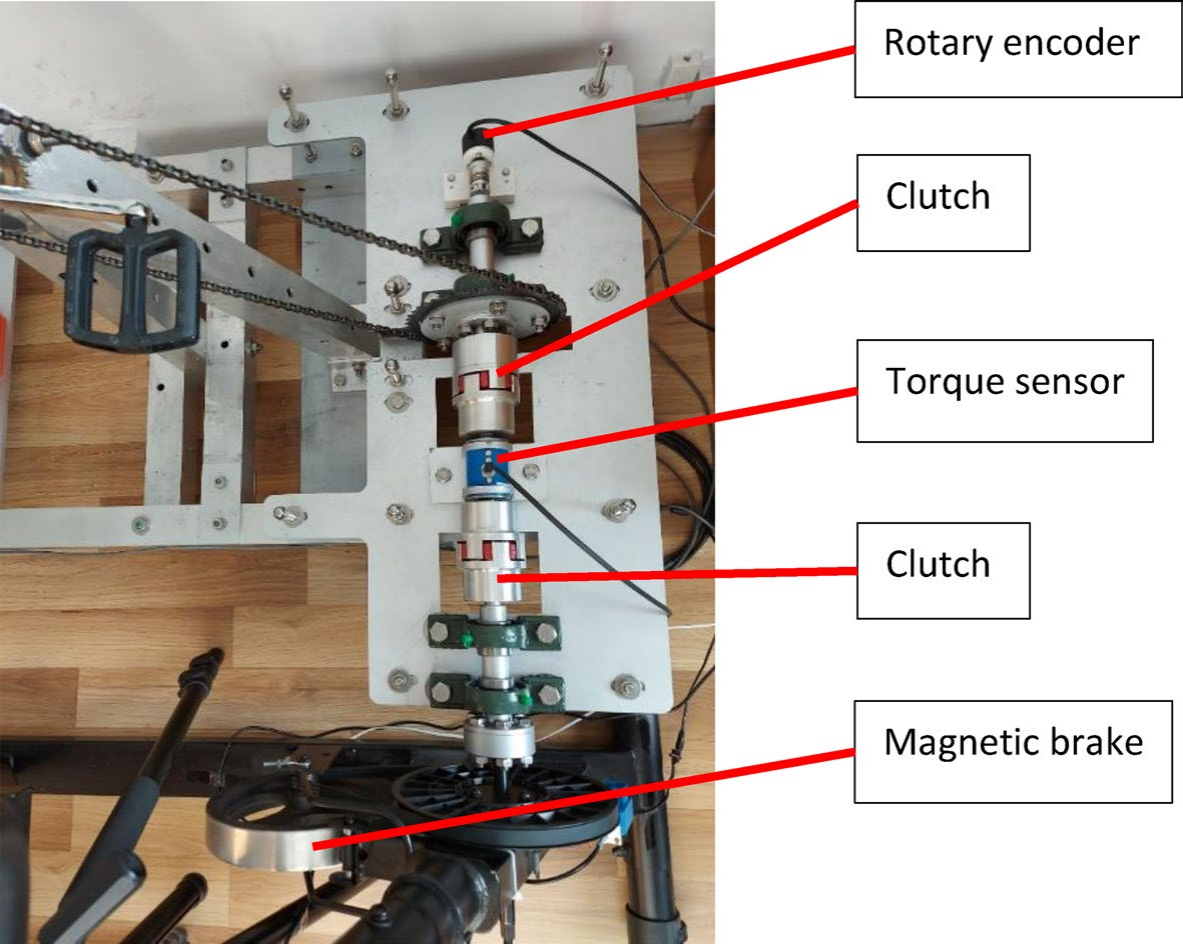
Track for measuring mechanical energy.

**Figure 5:**
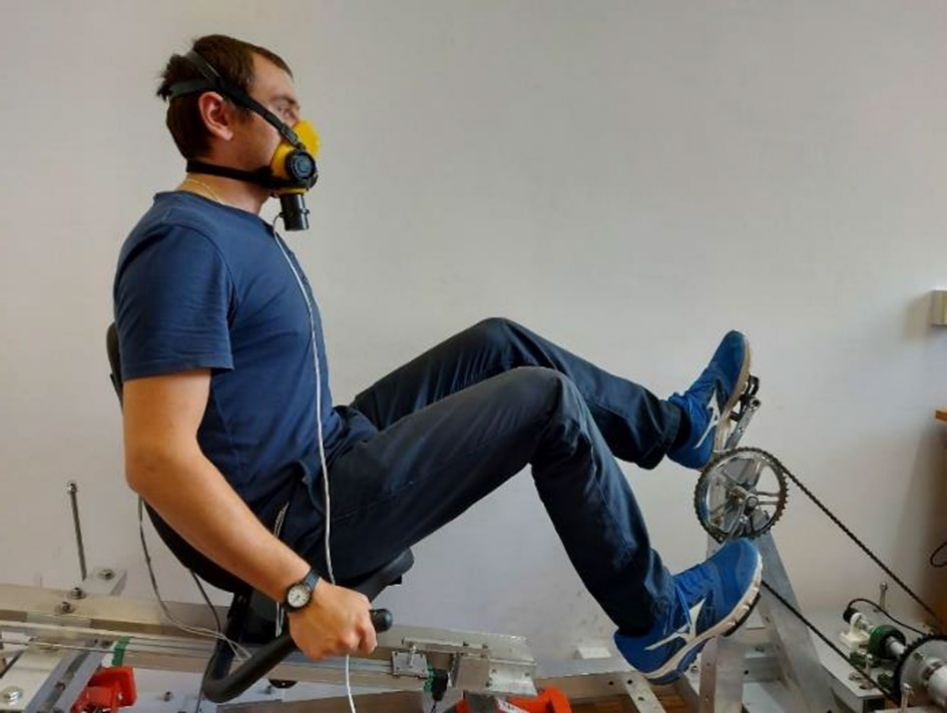
An example of a person on a test stand.

Figure 3 shows the test stand for dynamic testing and indicates the most important components of the stand. Figure 4 shows in detail the track for measuring mechanical energy. In turn, Fig. 5 shows the corresponding author during the demonstration of the test, wearing a half mask equipped with an airflow sensor. The participants have given informed consent for the publication of the image in an open access online publication.

#### Participants

A total of 36 tests were carried out. Healthy male students of Warsaw University of Technology took part in the dynamic research. The participants were 20.83 ± 0.17 (± SE) years old, 178.75 ± 1.18 cm height, weighed 72.97 ± 1.45 kg, and had a BMI of 22.84 ± 0.40 kg/m^2^. Exclusion criteria comprised: age below 19 and above 25, BMI above 30, female gender, active sports player (non-recreational), known chronic disorders that could be of a risk during physical effort as well as affect data collection and analysis. The number of tests was planned on the basis of experimental tests which were carried out before the main testing campaign. All participants were informed about the purpose of the study during the recruitment process and signed informed consent prior to the experimental protocol. All methods were carried out in accordance with relevant guidelines and regulations. All methods were approved by the Bioethics Committee at the Medical University of Warsaw, and their decision number KB/53/2022 of April 11, 2022, was obtained in order to conduct dynamic research. The participants’ consent to participate in the tests was given by a doctor on the basis of interviews, which took place immediately before the test. Participant data was anonymized and distribution was restricted. The tests were carried out at a load of 50 W, so no special medical protection was required during the tests.

Bicycles are generally used as an alternative means of transport mainly by relatively young people^33,34^, so the research involved student volunteers. Choosing students of similar age also has a positive effect on the stability of the drive source^35–38^, which is particularly important in assessing the efficiency of the human-mechanism system. Human performance changes with age, which is another variable, and these studies focused on the stability of the research environment. Therefore, when defining the research group, age was one of the key criteria. Only men were tested to make the research group as homogeneous as possible^39–41^.

#### Experimental procedure

**Figure 6:**
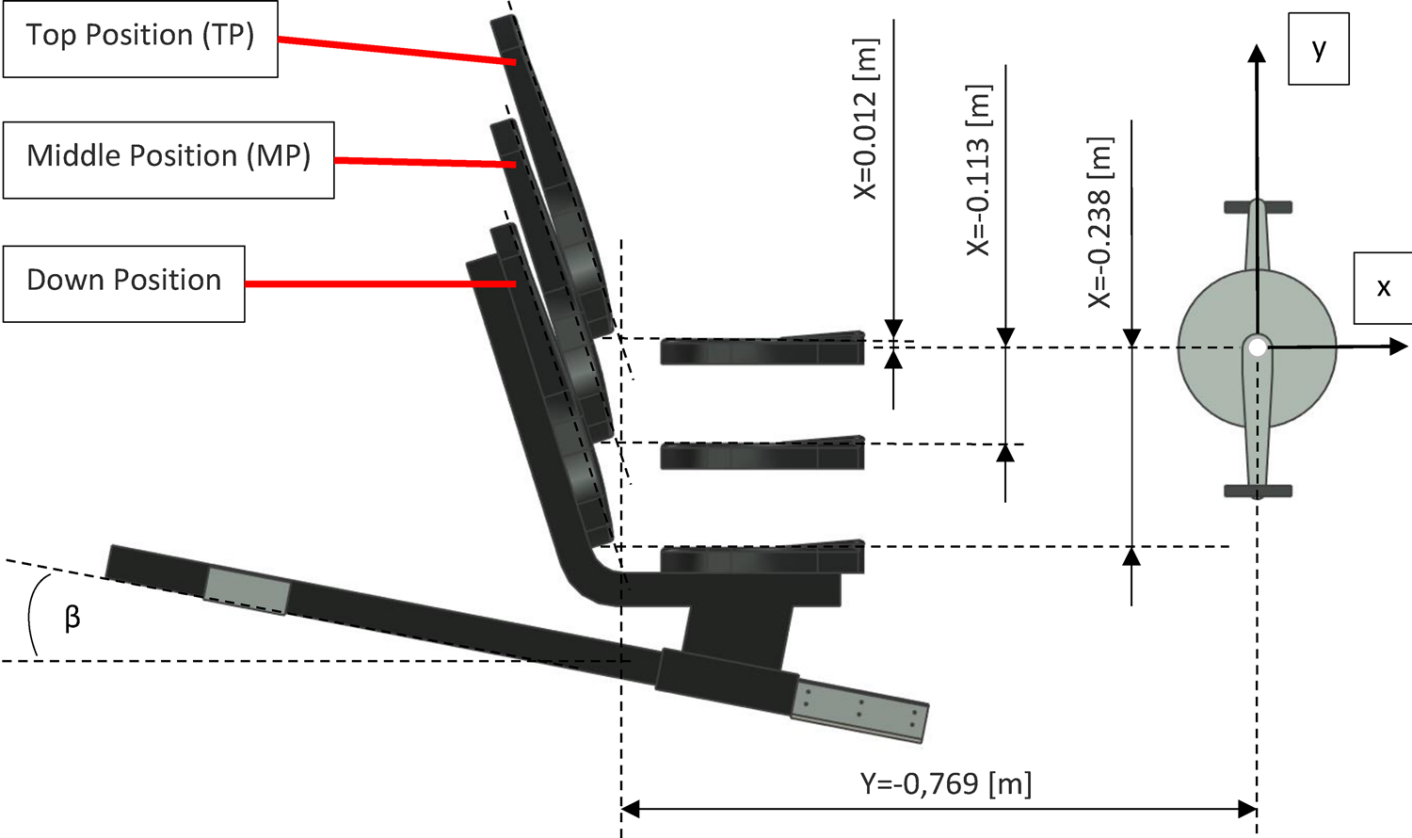
Position of the seat relative to the crankset for various scheduled seat test positions (assuming that m = 0 [m]).

Three positions of the seat relative to the crankset were tested: TP–top position, MP–middle position, DP–down position (see Fig. 6). Before the test, each participant individually set the horizontal distance of the seat (seat position, m) from the crankset. The height of the seat in relation to the crankset was set in predetermined positions—the sequence in which the tests were carried out on subsequent participants is presented in Table 1. It should also be mentioned here that the seat was mounted on a guide set at an angle of β = 15° to the horizontal plane, so sliding the seat substantially backward resulted in the height of the seat being raised. The inclined guide of the seat made it possible to maintain geometric similarity during the work of the skeleton between people of different heights.

**Table 1:** Data about the test sequence, the position of the seat during the test and user preference.

Test number	Test sequence			Seat position m [m]	User preference (best to worst)
	1 (P10)	2 (P12)	3 (P14)		
1	MP	TP	DP	0.094	TP, MP, DP
2	MP	DP	TP	0.171	MP, DP, TP
3	TP	MP	DP	0.193	MP, TP, DP
4	TP	DP	MP	0.246	TP, MP, DP
5	DP	TP	MP	0.225	TP, DP, MP
6	DP	MP	TP	0.216	MP, DP, TP
7	MP	TP	DP	0.157	MP, DP, TP
8	MP	DP	TP	0.260	MP, TP, DP
9	TP	MP	DP	0.203	TP, MP, DP
10	TP	DP	MP	0.180	MP, TP, DP
11	DP	TP	MP	0.219	MP, TP, DP
12	DP	MP	TP	0.219	TP, MP, DP
13	MP	TP	DP	0.212	MP, TP, DP
14	MP	DP	TP	0.212	MP, DP, TP
15	TP	MP	DP	0.212	TP, MP, DP
16	TP	DP	MP	0.223	TP, MP, DP
17	DP	TP	MP	0.291	TP, MP, DP
18	DP	MP	TP	0.218	MP, TP, DP
19	MP	TP	DP	0.100	MP, TP, DP
20	MP	DP	TP	0.173	MP, TP, DP
21	TP	MP	DP	0.119	TP, MP, DP
22	TP	DP	MP	0.293	TP, MP, DP
23	DP	TP	MP	0.162	MP, TP, DP
24	DP	MP	TP	0.201	TP, MP, DP
25	MP	TP	DP	0.194	TP, MP, DP
26	MP	DP	TP	0.226	TP, DP, MP
27	TP	MP	DP	0.138	MP, DP, TP
28	TP	DP	MP	0.201	TP, MP, DP
29	DP	TP	MP	0.151	TP, MP, DP
30	DP	MP	TP	0.161	TP, DP, MP
31	MP	TP	DP	0.217	MP, DP, TP
32	MP	DP	TP	0.193	MP, TP, DP
33	TP	MP	DP	0.166	TP, MP, DP
34	TP	DP	MP	0.211	TP, MP, DP
35	DP	TP	MP	0.204	TP, MP, DP
36	DP	MP	TP	0.168	TP, MP, DP

The study participants were divided into 6 groups. In other words, every 6 tests the test sequence is repeated (see Table 1). The research was planned in such a way as to be able to examine the impact of human fatigue as a function of the test sequence.

Dynamic tests were carried out for a power of 50 W so that the human load would be submaximal^42,43^. Experiment’s participants working at a low power made it possible to obtain relatively stable working conditions between different people, compared to a theoretical situation in which the research would be conducted at a higher power, e.g. 200 W. The choice of a low power was made due to the fact that people may have different levels of training and may react differently to physical effort. In addition, the 50 W load represents a very relaxed cycling experience. The load of 50 W partially eliminates the problem of changing efficiency depending on the cadence^44^. For small cadences and a 50 W load, the net efficiency is considered to be essentially constant, which favourably affects the overall test result by further limiting the variables. In addition, in the case of a power demand higher than 50 W, the vehicle can be supported by an electric motor, making sure not to overload the human body, whose regeneration may require more than 24 h. That is why considering the design of a personal everyday vehicle, the load of 50 W during the tests seems to be well matched.

**Figure 7:**
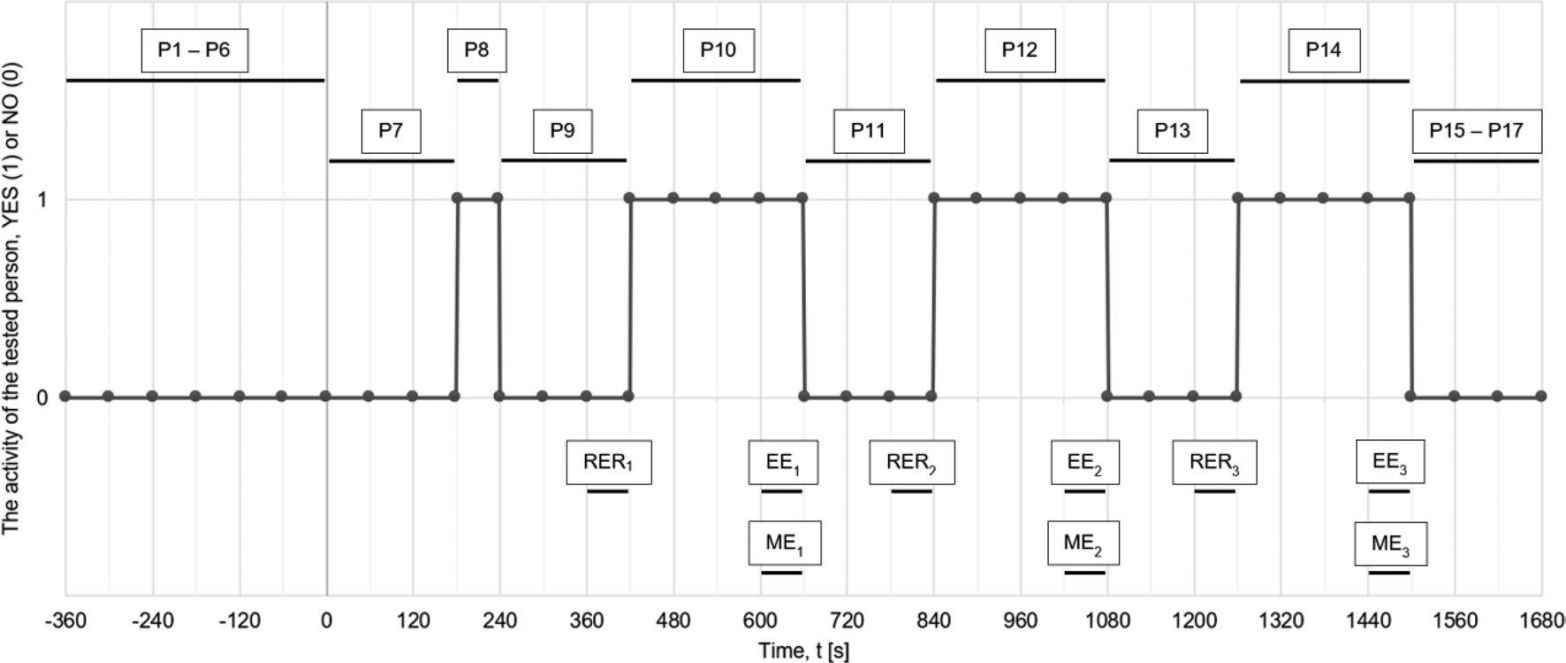
The test plan presented in the form of a graph.

The dynamic test procedure that was used during the tests is presented below and schematically shown in Fig. 7:

1. Conducting an interview by the doctor with the tested person regarding e.g. occurrence of diseases that may be contraindications to exercise. Assigning a working number to the tested person. Collecting data on the age, height and weight of the tested person.
2. Adjusting the seat to the middle position (MP).
3. Entering the data about the tested person into the computer controlling the magnetic brake and switching on the mode of maintaining the set power of 30 W.
4. The tested person sits on the seat and sets the distance m of the seat from the crankset.
5. Measurement of pulse and blood pressure using an external measuring device.
6. Connecting the pulse sensor to the tested person and putting on a half mask equipped with a flow sensor.
7. Adaptation of the subject’s breathing for 3 min.
8. Warm-up conducted at the power of 30 W for 1 min and at the same time the test person’s adaptation to the test stand.
9. A break of 3 min for the tested person. Increasing the load on the magnetic brake to 50 W. Setting the seat to the position in accordance with the planned sequence (TP or MP or DP—see Table 1).
10. Trial 1 performed by the tested person for 4 min.
11. A break of 3 min for the tested person. Setting the seat to the position in accordance with the planned sequence (TP or MP or DP—see Table 1).
12. Trial 2 performed by the tested person for 4 min.
13. A break of 3 min for the tested person. Setting the seat to the position in accordance with the planned sequence (TP or MP or DP—see Table 1).
14. Trial 3 performed by the tested person for 4 min.
15. Measurement of pulse and blood pressure using an external measuring device.
16. Disconnecting the measuring apparatus from the tested person.
17. Completion of the questionnaire by the tested person regarding the preferred position of the seat.

During the test, the screen in front of the test stand displayed messages regarding the activities that the tested person should perform, which was intended to precisely control the test schedule. During the exercise, the tested person held his hands on the handles next to the seat. During the breaks between period of exercise, the subject kept his feet on the floor. Changing the position of the seat (TP or MP or DP) was possible without getting up from the seat.

When processing the measured baseline data to determine the gross and net efficiencies, only specific study areas were analysed. The specific areas of study are shown in Fig. 7 and are RER, EE and ME, and their interpretation is given below:

- RER [J]–Resting Energy Rate—it is the resting energy that is used for life processes, measured just before starting the main exercise for 60 s,
- EE [J]–Energy Expenditure—it is the total energy expended by a person during the exercise, measured in the last 60 s of the exercise, when the person should have stabilized breathing in relation to the work they performed during the test,
- ME [J]–Mechanical Energy—it is the usable energy that can be used in transport, measured over exactly the same time interval of 60 s as EE.

The following basic data was recorded during the tests:

- Time, t [s]—controlled and measured using a National Instruments measuring card (USB-6251), data reading from all measuring devices was carried out every 0.015 s also using this measuring card,
- Angular position, φ [rad]—measured with Rotary Encoder (PIB406C-3600-G5-24-C, 3600 imp/rev),
- Torque (moment of force), M [Nm]—measured with Torque Sensor by NCTE AG (DFM22-250-S round shaft, 250 Nm),
- Pulses from the flow sensor, I [1]—measured with a sensor mounted in half mask, which was removed from the MWE-1 energy expenditure meter,
- Local seat position, k, l, m [m]—measured using 3 linear potentiometers, with the indications of the front and rear potentiometers being identical (k = l), because the seat inclination was not changed during the tests, the position of k and l was planned in advance (for TP the seat position was basically equal to k = l = 0.25 [m], for MP the seat position was basically equal to k = l = 0.125 [m], for DP the seat position was basically equal to k = l = 0 [m]),
- ECG signal of the tested person, ECG [V]—measured with the Analog Heart Rate Monitor Sensor (DFRobot Gravity with AD8232 heart rate monitor), which used 3 ECG Electrodes (Premier FS-RG1/10 from SKINTACT) glued to the chest,
- Temperature and air pressure, T_1_ [K], P_1_ [Pa]—measured using sensors dedicated to the Arduino system,
- Air humidity, f [%]—read by the tester from the weather station (Anytech DC102) before each exercise.

During the tests, a magnetic brake removed from the ZIPRO Recumbent Bike (“Glow” model) was used^45^. WATT program was used during the tests. It is a program that allows maintaining a constant set power—in other words, when the tested person changes the cadence, the system automatically changes the load so that the energy expenditure of the tested person is consistent with the settings. The power control function depending on gender, age, height and weight used by the magnetic brake is not known.

The basic data collected during dynamic tests were transformed into gross and net efficiency in accordance with the formulas presented in Section “Theory and calculations“.

Due to the amount of the collected data, a special program was used to automate the calculations in order to reduce the time needed for the analysis of the data collected from the dynamic tests. Finally, the gross and net efficiencies of the human-mechanism system were obtained from the dynamic tests. Statistical methods were used to analyse the calculated efficiencies.

#### Statistical analysis

Statistical analysis of the dynamic experiment data to find the optimal position of the seat related to the crankset was performed using Statistica v.13.1 software (Statsoft, PL). The results were presented as mean values and standard error. The normality of data distribution was assessed using Shapiro–Wilk test. In the analysis the data that did not have normal distribution were assessed using Kruskal–Wallis ANOVA (KW) as well as Dunn’s multiple comparison test and Mann–Whitney U test (UMW) for estimation of the differences between the groups within particular parameters. All the hypotheses evaluated used a significance level of 0.05.

## Theory and calculations

This section introduces the key calculations and transformations that form the basis of the theory for this research.

One of the objectives of the dynamic tests was to determine the gross and net efficiency. Below are the formulas used to determine these values based on the basic data (see Section “Experimental procedure“) collected during the tests.

Gross efficiency:

1 $${\eta }_{gross}=\frac{Mechanical\, Energy (ME)}{Energy\, Expenditure (EE)}*100\%$$

Net efficiency:

2 $${\eta }_{net}=\frac{Mechanical\, Energy (ME)}{Energy\, Expenditure \left(EE\right)-Resting\, Energy\, Rate (EE)}*100\%$$

Mechanical Energy:

3 $$ME=\int M(\varphi ) d\varphi$$

**Figure 8:**
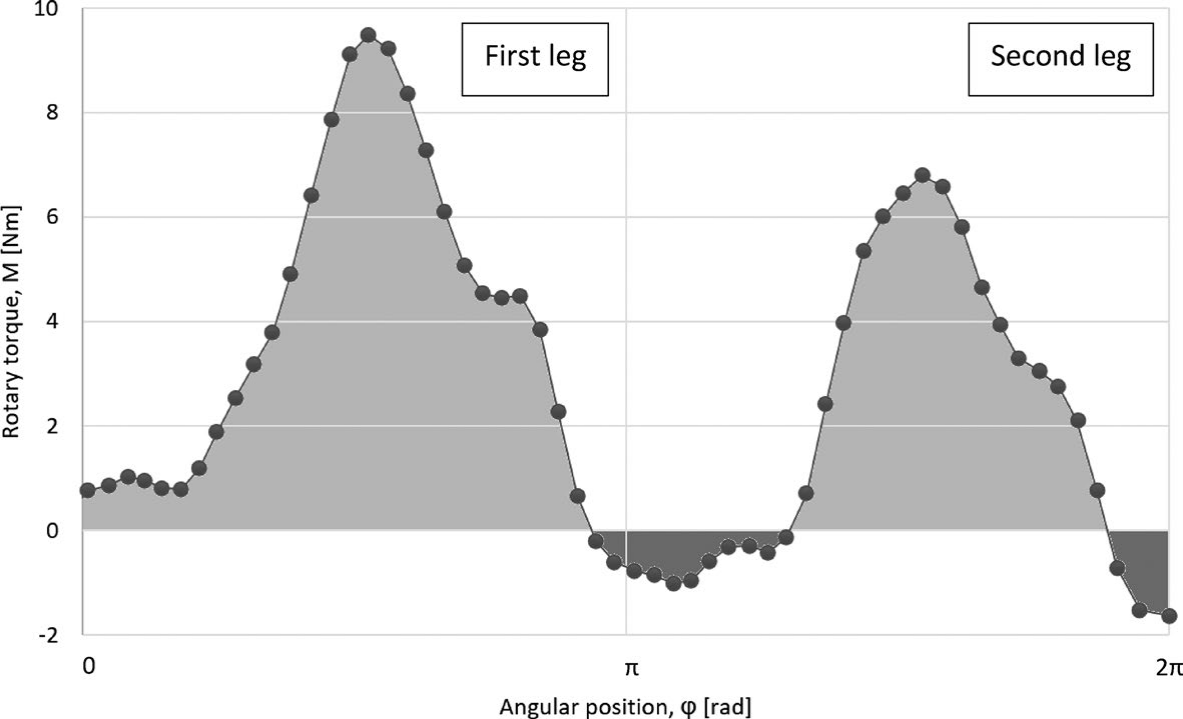
Diagram of M(φ), with negative energy visible.

Collected Mechanical Energy, ME [J], was considered over a time interval of 60 s (see Fig. 7). The integration was performed by the trapezoidal method. Locally, the energy was negative (Fig. 8), which resulted from a temporary interruption in the supply of energy by the tested person to the crankset. The reason for that situation was that the energy accumulated in the inertia of the measuring track generated a negative torque on the torque sensor—there was no freewheel installed on the power take-off shaft. It was assumed that negative energy ultimately assists in movement on the next work cycle of activating the other leg to produce mechanical energy and should therefore be considered positive energy. This negative energy should not be considered as a waste, because this energy allows the subject’s leg to be moved into the area when the next phase of work will be done—in other words, negative energy lifts the leg in the gravitational field. Negative energy did not occur for each of the tested persons, and if it did occur in any person, it did not appear at every turn of the crankset.

The starting torque for the entire measurement path, when the drive belt connected to the magnetic brake was unfastened, was 0.767 [Nm]. This braking torque includes bearing resistance in the crankcase, chain movement resistance, bearing resistance in the measuring track and magnetic brake, and clutch movement resistance. In the analysis, the starting torque was not subtracted from the torque that was used to determine the mechanical energy, because it was considered that some braking torque in the personal vehicle would also be present.

The formulas for calculating the EE and RER are essentially the same, but they are calculated over different time zones. Nevertheless, both of these values are calculated on a time interval of 60 s. Based on the literature^46^, it was assumed that the examined person obtains a stable breath after 3 min, which is why the last 60 s of the exercise, which lasted 4 min, were selected for analysis. The formulas for this data allow conversion of the successive pulses coming from the flow sensor into the amount of energy expended during the actual test (EE) or in the final rest phase before the actual test (RER).

Based on the basic data measured during the test, both EE and RER can be determined according to the formulas below.

Speed of the flow sensor turbine:

4 $$\omega =\frac{{I}_{n+1}-{I}_{n}}{{t}_{n+1}-{t}_{n}}$$

where t_n_ and t_n+1_ are successive minimum time intervals of 0.015 s, while I_n_ and I_n+1_ are pulses registered over the same time interval. The higher the flow velocity, the greater the pulse difference between successive measurement points. When the subject was exhaling, the turbine speed was 0 [imp/s], because the flow sensor valve was used to close the air access to the turbine, and the valve allowing air to be released from the lungs was opened without moving the turbine backward. In the case of inspiration, the valves were switched to the opposite state, which resulted in the registration of incrementally successive impulses.

The flow sensor was calibrated with the use of a standard flow meter, which allowed determination of the formula that is used to convert the turbine speed of the flow sensor into air flow. A linear function with a slope of 0.16 [1] permitted calculation of the air flow.

Air flow:

5 $${V}_{1}=0.16*\omega$$

**Figure 9:**
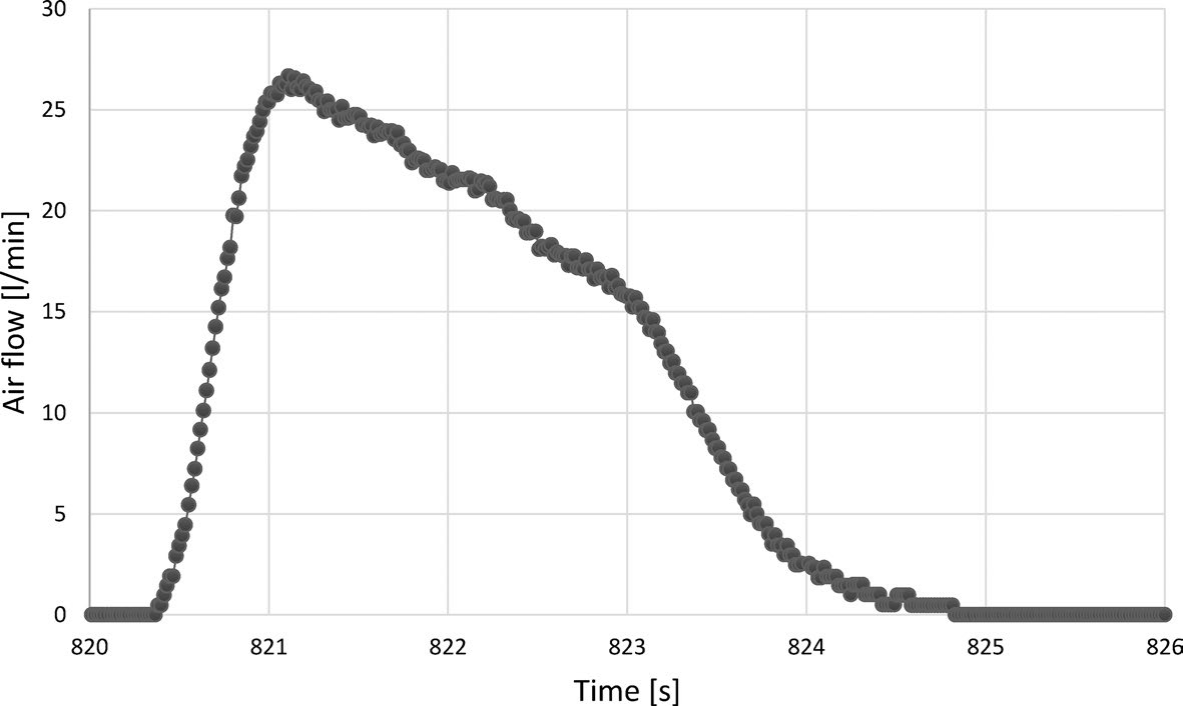
Airflow in one full breath during exercise.

The air flow was expressed in litres per minute [l/min], which ultimately enabled the use of formulas to determine the amount of energy expended. An example graph showing the flow per breath is shown in Fig. 9. In order to calculate the amount of energy expended, the flow should be transformed into the so-called standard conditions (STPD—Standard Temperature Pressure Dry)^47^. Dry means the complete absence of water vapor in the air.

During the tests, temperature (297.51 ± 0.16 K (± SE)) and pressure (100386.3 ± 107.7 Pa) were recorded in parallel with other data. Air humidity was checked once before starting the test. During subsequent tests, the humidity of the air did not change significantly (38.8 ± 1.1%); moreover, the change in volume was very small due to the change in humidity, so the correction of the air volume was not taken into account. Volume correction was performed using “ideal gas law”. Taking into account the lack of correction of the volume by air humidity, the designation STP(D) was introduced.

Air flow STP(D):

6 $${V}_{2}={V}_{1}*\frac{{P}_{1}}{{P}_{2}}*\frac{{T}_{2}}{{T}_{1}}$$

The analysis assumes that the standard temperature T_2_ is equal to 273 [K], and the standard pressure P_2_ equals 101300 [Pa].

With this data, it is possible to determine the amount of energy expended by a person.

Energy expended by the tested person:

7 $$E=0.210*{V}_{2}$$

The factor 0.210 in the formula is related to the amount of oxygen in the inhaled air. The energy expended by humans E [kcal/min] was converted into SI units.

Energy expended by the tested person expressed in SI units:

8 $${EE}_{t,s}=\frac{4184*E}{60}$$

The energy expended by humans EE_t,s_ [J/s] was calculated for each i-th measurement interval. Time was recorded cumulatively, hence the formula for the energy portion in the i-th measurement has the following form.

A portion of energy over the minimum recorded time interval:

9 $${EE}_{i}={EE}_{t,s}*\left({t}_{n+1}-{t}_{n}\right)$$

where t_n_ [s] and t_n+1_ [s] are the consecutive recorded times.

Finally, Energy Expenditure, EE [J] is the sum of all i-th portions of energy occurring in the time interval t = 60 s in the final phase of the exercise:

10 $$EE=\sum_{0}^{t}{EE}_{i}$$

In turn, Resting Energy Rate, RER [J] is the sum of all i-th portions of energy occurring in the time interval t = 60 s in the final phase of rest:

11 $$RER=\sum_{0}^{t}{EE}_{i}$$

During the research, the heart rate was monitored to verify whether the exercise load did not cause the person to go beyond the submaximal area. To determine the human heart rate, the ECG signal [V] was analysed to detect the characteristic needles describing the work of the heart^48–50^. Knowing the time interval at which the next heartbeat occurred, it was possible to determine the pulse.

**Figure 10:**
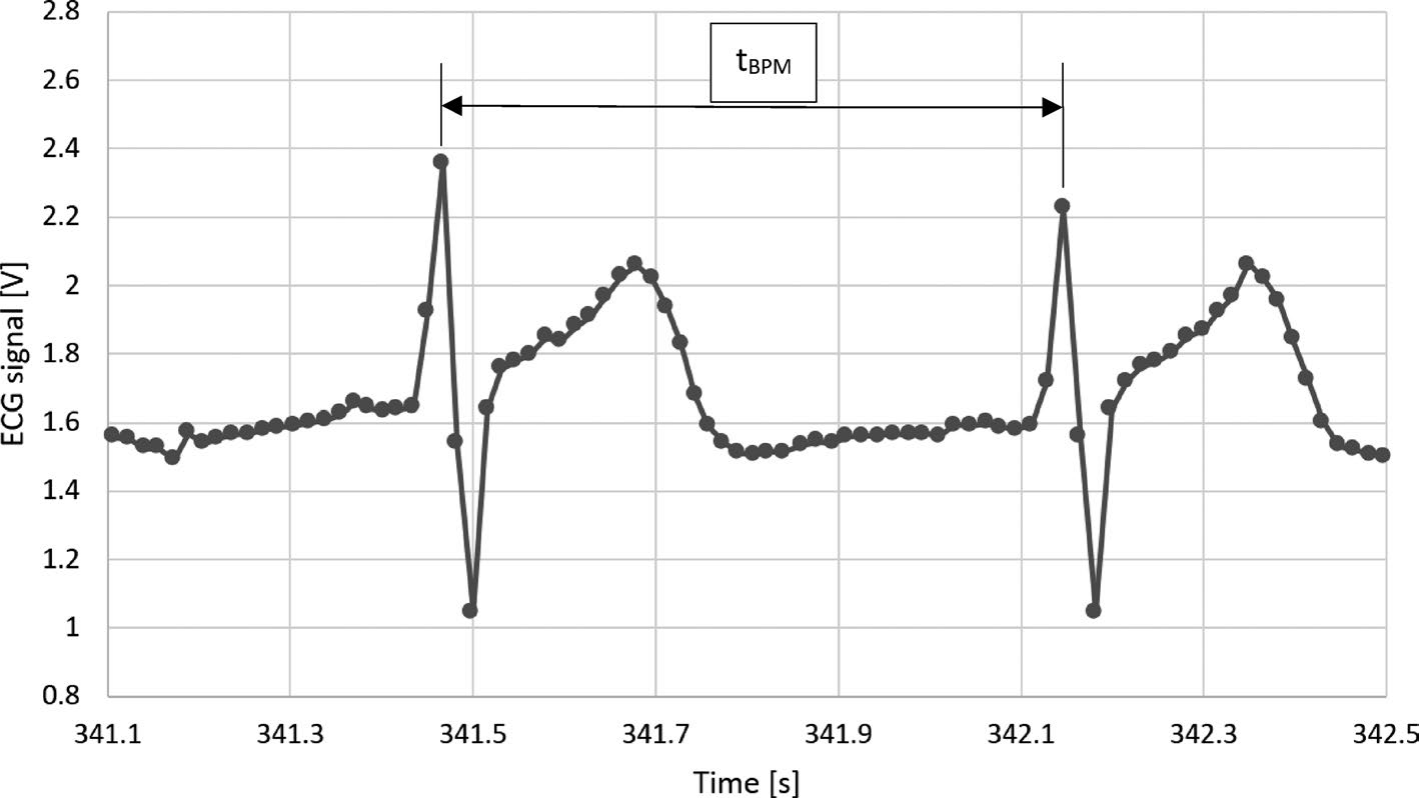
Example fragment of the ECG signal.

Pulse rates, in beats per minute (BPM):

12 $$BPM=60*\frac{1}{{t}_{BPM}}$$

where t_BPM_ [1/min] is the time between two characteristic points on the ECG signal (see Fig. 10).

The exact position of the seat (x, y) during the test can be calculated from the formulas below. These formulas take into account the position of the jacks (TP or MP or DP), the essentially horizontal distance m of the seat from the crankset and the position correction resulting from the inclination of the guide to the angle β = 15°.

The exact position of the seat during the individual test—coordinate x:

13 $$x=X+m*{\text{cos}}\beta$$

The exact position of the seat during the individual test—coordinate y:

14 $$y=Y-m*{\text{sin}}\beta$$

where X and Y are constants resulting from the position of the jacks. For the Top Position X = 0.012 [m] and Y = − 0.769 [m], for the Middle Position X = − 0.113 [m] and Y = − 0.769 [m] and for the Down Position) X = − 0.238 [m] and Y = − 0.769 [m].

## Results

### Dynamic research

Dynamic tests were carried out to determine what position of the seat relative to the crankset would maximize the efficiency of the human-mechanism system. In addition, ergonomic aspects were evaluated during these tests.

Table 1 contains information about:

- Test sequence,
- Position of the seat during the test,
- Preferred seat position from the most preferred to the least preferred.

### Statistical analysis

**Figure 11:**
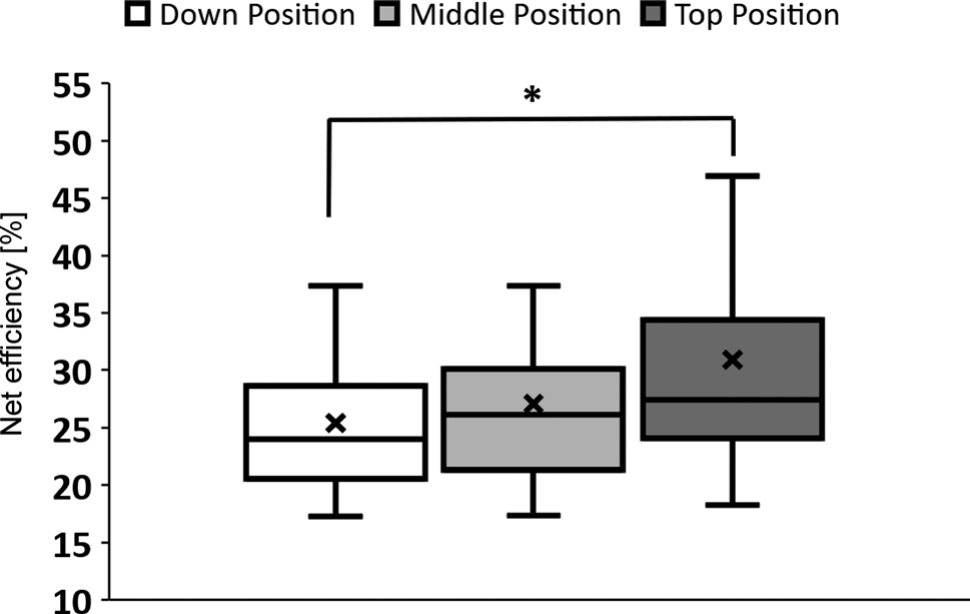
Net efficiency of different seat positions (*p < 0.05, Dunn Test – Down Position vs. Top Position).

In the analysis of the data obtained during the research the statistically significant differences were found in net efficiency (η_net_) between the Down, Middle and Top Positions of the seat (H_(2,108)_ = 6.87; *p* < 0.03, Kruskal–Wallis ANOVA) taking into account the measurement during the last minute of particular trial. The post-hoc examination showed greater efficiency for the Top Position (η_net_ = 30.88 ± 1.85%) in comparison to the Down Position (η_net_ = 25.42 ± 1.16%, *p* < 0.05, Dunn Test; *p* < 0.01, UMW test), but not to the Middle one (η_net_ = 27.08 ± 1.19%, *p* > 0.05 Dunn Test) (Fig. 11). As in the detailed examination no statistical significance was shown between net efficiency for a particular seat position and different trial number (procedure points P10, P12, P14) during the experimental cycle (Down Position: H_(2,36)_ = 1.1; *p* > 0.05, KW; Middle Position: H_(2,36)_ = 3.25; *p* > 0.05, KW; Top Position: H_(2,36)_ = 0.04; *p* > 0.05, KW), so the trials were pooled for analysis up to 108.

## Discussion

Statistical analysis of the data obtained from the dynamic tests showed that the Top Position of a seat is the best. In the case of gross efficiency, statistical analysis showed a similar nature of the graph to the one for net efficiency, but did not show a statistical significance between seat positions. No significant differences between height of the tested person and the efficiency were noticed. User preference does not coincide with the maximum efficiency between chair settings—in other words, the subject was unable to judge which setting gave the greatest efficiency.

Below, an exemplary, simple optimization of the seat for a personal vehicle moving at a general speed of about 15 km/h was made. Table 2 shows the estimated share of other factors influencing the position of the seat. Estimating the exact weights requires determining all the parameters in which the personal vehicle will operate, whether it will be supported by a motor or not, etc. In the case of breaking the speed record, it is necessary to reduce the resistance to movement, including the air, but in everyday use and when electric support maybe included it is not so important. The analysis assumed constant weights equal to 25% for each of the criteria; however, their precise definition requires a broad analysis of personal vehicle utility parameters. The evaluation scale was adopted from 0 to 10. The visibility of the surroundings and vehicle motion resistance were assessed in a linear manner, i.e. 10 points for the best solution, 5 points for the average and 0 points for the worst one. On the other hand, the efficiency and preferences of use were assessed based on the data obtained from the conducted research. Taking into account the four criteria for optimizing the values in Table 2, it appears that the Top Position of the seat is the best option. It should be noted that the Top Position has a significant advantage over the other positions.

**Table 2:** Optimization of the seat position for personal vehicle.

	Weight (%)	TP	MP	DP
Surround visibility	25%	10 points	5 points	0 points
PV motion resistance	25%	0 points	5 points	10 points
Efficiency	25%	10 points for 30.88%	3.04 points for 27.08%	0 points for 25.42%
User preference	25%	5.56 points for 20 indications out of 36	4.44 points for 16 indications out of 36	0 points for 0 indications out of 36
SUM	100%	63.9%	43.7%	25.0%

## Conclusions

Dynamic studies have shown that higher seat positions result in a statistically higher efficiency. When designing a personal vehicle, it should be borne in mind that efficiency is not the only factor to be considered. When it comes to a personal vehicle for generally low-speed mobility, the Top Position of the seat is also the best solution. In addition, it should be remembered that changing the target group of personal vehicle users may change the optimal position of the seat in terms of efficiency, and in addition, the power of motors supporting the movement of the vehicle is crucial in these analyses.

## Data Availability

Detailed data about the participants of the research are not publicly available due to the risk of identification. The raw baseline data collected during the tests and the calculated gross and net efficiencies are available from the corresponding author on reasonable request.
